# Does Financial Technology Improve Health in Asian Economies?

**DOI:** 10.3389/fpubh.2022.843379

**Published:** 2022-02-14

**Authors:** Ran Jing, Yechi Ma, Liangyu Zhang, Muhammad Hafeez

**Affiliations:** ^1^School of Finance, Changchun University of Finance and Economics, Changchun, China; ^2^School of Business, Northeast Normal University, Changchun, China; ^3^Faculty of Management and Administrative Sciences, University of Sialkot, Punjab, Pakistan

**Keywords:** FinTech, health, Asia, GMM, ATMs

## Abstract

The progress of the health sector in a sustainable manner is crucial for the development of human capital, a significant and vital driver of economic growth. Hence, we aim to investigate the impact of FinTech on health outcomes in Asian economies over the period 2007–2019. The empirical estimation of the study is based on the 2SLS and GMM techniques. The outcomes confirmed the negative impact of ATMs and Debit cards on the infant mortality rate in both 2SLS and GMM models. Whereas, ATMs and Debit cards positively impact the life expectancy of people living in Asian economies irrespective of the estimation technique. Similarly, the association between the Internet and infant mortality rate is negative; whereas, this association is positive in the context of the Internet and life expectancy both with 2SLS and GMM. From these findings, we can confirm that the amalgamation of technology and the financial sector helps to improve health outcomes in Asian economies. Therefore, the integration of FinTech into the health sector should be part and parcel of every health policy in emerging Asian economies.

## Introduction

The sustainable growth of the healthcare system is a stimulating determinant of human capital and a fundamental feature of economic development. A large body of prevailing literature denoted that healthier workers or citizens are more mentally stable, more energetic, and more productive, and during illness, their chances to get absent are smaller as compared to unhealthy workers (1, 2). Thus, the sustainability of the healthcare sector is important for social cohesion, labor market efficiency, and macroeconomic stability (3). Owing to the utmost importance of the health care system, policymakers are researchers, and health economists are very keen to investigate several mechanisms to increase the sustainability and survival of the health care system.

The speedy revolution of the industrial sector has transformed the value of information technology into the business sector that facilitates the information management of healthcare and healthcare systems (4). More specifically, financial technology (FinTech) has transformed the processes of business by integrating finance toward technological advancements and innovations. Access to digital money and electronic finance services has interchanged the digital divide as a significant and unintended barrier to lower-income individuals to contribute such technologies that lead to good quality health outcomes. Due to the high cost attached with financial intermediation, FinTech has guaranteed to enhance the welfare of consumers by overwhelming the financial contracting divisions (5). Regardless of the extensive use of the terminology, its explanation differs in literature. The financial stability board establishes universal recognitions, which explains FinTech as technology-based financial innovation that results in new products, processes, applications, or business models associated with material effects on financial institutions and markets, and the endowment of finance-related services.

FinTech significantly covers credit services, deposits, capital-raising, settlement services, digital payments, management services, and insurance that also streamlines and facilitates healthcare processes by reducing the expenses of financial services associated with the conventional financial setup (6–8). Through blockchain technologies, machine learning, artificial intelligence, mobile payments, and robotic devices of investment, FinTech boosts improvements to the healthcare system by reducing inefficiencies related to payment plans (9, 10). FinTech provides a solution for financing issues that reduce financial exclusion and income inequality and enable moderate and low-income individuals to access and afford healthcare services (11). Literature denotes that FinTech development contributes significantly to mitigating poverty (12, 13).

FinTech are thoroughly illuminating the socio-economic features because of their impartiality from the formally developing an association of lending and borrowing (14). Subsequently, borrowing has become easier due to the FinTech approach. Thus, the burdens and challenges associated with healthcare payments have been condensed. The FinTech approach simplifies and expedites the payment process of healthcare for individuals, and mobile-based accounts for health savings and low-interest loans have raised the affordability of health services for middle and lower-income segments of the society (15). Regardless of the direct impact of blockchain-related FinTech platforms on patients' and consumers' healthcare comfort, there is a shortage of empirical evidence on how FinTech contributes to the sustainability of the healthcare system. We are also exploring the role of ICT as a control variable in reinforcing the health-FinTech nexus that is more important with FinTech. Based on the previous studies, we proclaim that the maturity of ICT may catalyze environmental sustainability, social cohesion, and economic growth (16). ICTs and FinTech empower firms to be creative at a fundamental level and enhance the delivery level of healthcare services (17). Likewise, FinTech reduces carbon emissions, raises renewable energy consumption, and trade openness (18, 19). In the perspective of healthcare development, FinTech adopts healthcare expenditures as a transmitting channel to upgrade the system of healthcare in the economy (18). Thus, both ICT and FinTech support countries in achieving sustainable competitiveness.

The endogenous growth theory denotes technology and FinTech as imperative determinants of economic development (20). In view of the Solow growth model, capital and labor cannot enhance economic growth without technology transfers and technological spill overs (21). In the presence of FinTech, technology moderates asymmetry information between borrowers and lenders (22). In the FinTech context, machine learning and big data algorithms permit borrowers to get an easy calculation of credit, allow them to track their investments, and provide them financing opportunities that create trustworthy association among FinTech investors (23). FinTech competes efficaciously with incumbents with limited resources through affordable, faster, more flexible, and less complex financial resources. Based on disruption theory, it is assumed that FinTech targeted consumers first and then captivated most customers after attaining traction.

Emerging literature has confirmed that digital financial inclusion helps in improving the welfare of households by enhancing financial resilience and security and by reducing their mental stress (24, 25). Digital financial inclusion contributes to boosting the well-being and health of households through various measures, such as providing them easy access to insurance-related facilities, supporting households from getting rid of coping strategies, and offering them mental satisfaction (26). Ajefu et al. (24) reported that digital financial inclusion enhances the freedom of households by offering them services for managing their livelihoods. The literature further indicates that digital financial inclusion promotes financial capability and health by reducing the level of stress, increasing the stability of finance, and improving the health conditions of households. Huang et al. (27) report that digital financial inclusion results in promoting economic welfare in rural areas of China.

The present study fills the vacuum by investigating the impact of FinTech expansion on the sustainability of healthcare performance. The study will moderate the role of ICT as a control variable in the FinTech-health framework. Existing literature on the association between FinTech and healthcare sustainability is very limited. Deng et al. (14) investigated the association between sustainable performance and FinTech at a provisional level in the case of China. By further prolonging the existing literature, our study examines the unexplored impact of FinTech on healthcare sustainability in the case of the Asian region over the period 2008–2019. This study also contributed to the existing literature by investigating the moderating contribution of ICT on the nexus between FinTech and healthcare performance. Moreover, the study utilized the 2SLS approach and GMM approach to capture the issue of endogeneity. This study highlights the significance of FinTech in determining people's health, and the study will help policymakers design more sustainable health-related policies. Furthermore, the study provides new direction to upcoming researchers for digging the role of FinTech in enhancing the well-being of the economy. The current study results will help academia, development economists, central banks, and global organizations. The rest of the study is organized as follows. Section Model and Methods gives the model, methods, and data description. Section Results and Discussion analyses the empirical results, and section Conclusion and Implications concludes the study.

## Model and Methods

The connections between the group, family, individual financial well-being, and population health are considerable, complex, and growing. FinTech creates direct and predictable consequences that outcome in reduced population health. Latest years, scholars have assessed the health benefits of FinTech, especially in the COVID pandemic (28–30). Digital innovation of the financial sector has made the patients capable of paying their health expenditures in an emergency by reducing costs, which in turn facilitates healthcare systems. FinTech is also facilitated society by reducing income inequality, providing better healthcare services to deprived income groups, and improving the financial sector performance of the healthcare industry. Our study follows the latest empirical studies (28, 31). To detect the impacts of FinTech on health outcomes; we have used the following panel model forms:


(1) $$\begin{array}{l} Health_{it}=\;\varphi_{0}+\;\varphi_{1}Fintech_{it}+\varphi_{2}GDP_{it}+\varphi_{3}HE_{it}\\ \;+\varphi_{4}Internet_{it}+\varphi_{5}FDI_{it}+\alpha_{i}+\varepsilon_{it} \end{array}$$

Where are the health outcomes that depend on financial technology (*FinTech*), GDP growth (GDP), health expenditure (HE), internet users (Internet), and foreign direct investment (FDI)? FinTech contributes to public health, thus estimates of φ_1_ is expected to be positive. As for the effects of the control variables, health expenditure, GDP, HE, Internet, and FDI could be positive on health outcomes. In the panel model, α_*i*_ is an unobserved individual-country effect, while ε_*it*_ is the error term, but i and t represent country and time period, respectively. While *Health*_*it*−1_ is a lagged level of health outcomes in equation (2). The extended panel model is:


(2) $$\begin{array}{l} Health_{it}=\;\varphi_{0}+\lambda_{1}Health_{it-1}+\;\varphi_{1}Fintech_{it}+\varphi_{2}GDP_{it}\\ \;+\varphi_{3}HE_{it}+\varphi_{4}Internet_{it}+\varphi_{5}FDI_{it}+\alpha_{i}+\varepsilon_{it} \end{array}$$

Equation (1) can be estimated using numerous panel data methods. These methods include the pooled OLS, random effects (RE), fixed effects (FE), and instrumental variable methods such as two-stage least squares (2SLS). The classical estimation method such as FE and RE provide biased and inconsistent estimates due to endogeneity problems. We can employ the 2SLS approach in the basic model to address the endogeneity problem. We employ a GMM approach for panel equation (2) developed by Arellano and Bond (32), which can eliminate the problem of autocorrelation, heteroskedasticity, and endogenous variables. The GMM is an ideal approach for empirical studies with a small number of data periods and a large number of cross-sections (33). As our panel data contains 18 economies and a comparatively small time period of 13 years (from 2007 to 2019) and infers that GMM is an appropriate econometric method. Thus, to solve the problems of endogeneity, autocorrelation, heteroscedasticity, and omitted variable bias, this study used 2SLS, and GMM approaches.

### Data

The study aims to explore how FinTech contributes to improving the health performance in Asian economies over the period 2007–2019. Table 1 provides detailed information regarding descriptive statistics, the definition of variables, and sources of data. Table A1 provides the detail of selected countries. Health performance is a dependent variable that is measured by two proxies, such as life expectancy at birth in total years and infant mortality rate per 1,000 live births. The focused variable FinTech is also measured by two proxies, namely ATMs per 100,000 adults and debit cards in percent above 15 years of age. GDP growth (in annual percentage), current health expenditure (in the percentage of GDP), internet usage (in the percentage of population), and FDI (net inflows in percent of GDP) have been used as control variables in the regression. The ATMs and debit cards data are taken from the IMF, while data on all other variables have been extracted from the World Bank. Descriptive statistics are also described in Table 1. The mean of LE, IM, ATMs, Debit, GDP, HE, Internet, and FDI are 72.43 years, 20.71, 53.03, 30.71, 5.384, 4.336, 36.76, and 4.468%, respectively.

**Table 1:** Descriptive statistics and data sources.

**Variables**	**Mean**	**Std. Dev**.	**Min**	**Max**	**Definitions**	**Sources**
LE	72.43	4.478	64.42	83.49	Life expectancy at birth, total (years)	WDI
IM	20.71	15.74	2.100	74.10	Mortality rate, infant (per 1,000 live births)	WDI
ATMs	53.03	66.49	0.497	296.0	ATMs per 100,000 adults	IMF
Debit	30.71	24.03	0.987	95.48	Debit card (% age 15+)	IMF
GDP	5.384	2.858	−7.800	17.29	GDP growth (annual %)	WDI
HE	4.336	1.384	2.275	8.510	Current health expenditure (% of GDP)	WDI
Internet	36.76	27.45	1.000	96.15	Individuals using the Internet (% of population)	WDI
FDI	4.468	6.999	−37.15	43.91	Foreign direct investment, net inflows (% of GDP)	WDI

## Results and Discussion

The study uses two proxies to measure health performance: life expectancy and infant mortality rate. Table 2 displays the empirical findings of FinTech-life expectancy nexus, while Table 3 demonstrates the findings of FinTech-infant mortality nexus. The study adopted 2SLS and GMM approaches for empirical investigation. For capturing the contribution of FinTech, the study is using two proxy measures, namely ATMs and Debit cards. Two separate models have been regressed incorporating the impact of ATMs and Debit cards on health outcomes. In Table 2, Column 1 displays the outcome of 2SLS model for ATMs and infant mortality nexus, column 2 provides findings of 2SLS model for capturing the impact of debit cards on infant mortality rate, column 3 report the findings of ATMs impact on infant mortality rate for GMM model. In contrast, column 4 displays the outcome of the impact of debit cards on the infant mortality rate for the GMM model.

**Table 2:** FinTech and infant mortality (2SLS and GMM).

	**2SLS**		**2SLS**		**GMM**		**GMM**	
	**(1)**		**(2)**		**(3)**		**(4)**	
	**Coef**.	**t-stat**	**Coef**.	**t-stat**	**Coef**.	**t-stat**	**Coef**.	**t-stat**
L.IM					0.941^***^	(7.520)	0.952^***^	(4.130)
Atms	−0.588^***^	(3.650)			−0.520^**^	(2.270)		
Debit			−0.400^***^	(6.790)			−0.401^*^	(1.840)
GDP	−0.263	(0.930)	−0.133	(0.900)	−0.007^***^	(2.710)	−0.009^***^	(3.410)
HE	−2.598	(1.520)	−3.964^***^	(3.880)	−0.042^**^	(2.370)	−0.009	(0.580)
Internet	−0.355^**^	(2.340)	−0.008	(0.240)	−0.006^***^	(7.750)	−0.003^***^	(4.810)
FDI	−0.169	(1.170)	−0.184^***^	(2.690)	−0.002	(1.440)	−0.002^*^	(1.790)
Constant	−9.726^***^	(4.220)	5.412^***^	(3.710)	7.161^***^	(6.140)	3.164^*^	(1.800)
Observations	234		234		198		198	
Number of Country	18		18		18		18	
Sargan-test					0.356		0.578	

*T-stat in parentheses*.

*** *p < 0.01*,

** *p < 0.05*,

* *p < 0.1*.

**Table 3:** FinTech and life expectancy (2SLS and GMM).

	**2SLS**		**2SLS**		**GMM**		**GMM**	
	**(1)**		**(2)**		**(3)**		**(4)**	
	**Coef**.	**t-stat**	**Coef**.	**t-stat**	**Coef**.	**t-stat**	**Coef**.	**t-stat**
L.LE					0.924^***^	(4.630)	0.927^***^	(6.290)
Atms	0.134^***^	(4.070)			0.145^*^	(1.710)		
Debit			0.091^***^	(8.150)			0.078	(0.140)
GDP	0.059	(1.020)	0.029	(1.030)	0.018^**^	(2.200)	0.008^**^	(2.180)
HE	0.008	(0.020)	0.302^*^	(1.670)	0.023	(1.020)	0.022	(0.910)
Internet	0.059^*^	(1.920)	0.020^***^	(2.950)	0.001	(1.350)	0.001^*^	(1.730)
FDI	0.045^*^	(1.720)	0.036^***^	(2.740)	0.003^*^	(1.900)	0.003^*^	(1.750)
Constant	6.974^***^	(6.669)	7.226^***^	(9.210)	5.669^***^	(5.910)	5.410^***^	(5.680)
Observations	234		234		198		198	
Number of Country	18		18		18		18	
Sargan-test					0.546		0.329	

*T-stat in parentheses*.

*** *p < 0.01*,

** *p < 0.05*,

* *p < 0.1*.

In Table 2, the findings of 2SLS models display that ATMs and Debit cards exert a significant and negative impact on infant mortality rate, confirming that improvement on FinTech trends to reduce the infant mortality rate in Asian economies. Coefficient estimates reveal that a 1 percent upsurge in ATMs tends to decline infant mortality rate by 0.588 percent, while a 1 percent upsurge in Debit cards results in a declining infant mortality rate by 0.400 percent. Similarly, findings of GMM models demonstrate that FinTech contributes significantly in reducing infant mortality rate, hence improving health outcomes in Asian economies. Coefficient estimates reveal that 1 percent increase in the use of ATMs reduces the infant mortality rate by 0.520 percent, and a 1 percent increase in debit cards tends to reduce the infant mortality rate by 0.401 percent. In terms of control variables, findings infer that GDP, health expenditures, and FDI tend to reduce the infant mortality rate in two regression models, while the Internet results in reducing infant mortality in three regression models, confirming the improvement of health performance in Asian economies.

As described earlier, health outcome is measured by life expectancy as well. Table 3 displays the coefficient estimates representing the impact of FinTech on life expectancy in the case of 2SLS and GMM models. In Table 3, column 1 displays the impact of ATMs on life expectancy under the 2SLS model, and column 2 provides findings of the 2SLS model measuring the impact of debit cards on life expectancy, column 3 infers the impact of ATMs on life expectancy under GMM approach, while column 4 delivers the GMM findings capturing the impact of debit card on life expectancy. Findings of the 2SLS model reveal that ATMs and Debit cards exert a significant and positive impact on life expectancy, revealing that due to improvement in FinTech, health performance improves in Asian economies. Coefficient estimates reveal that a 1 percent upsurge in the use of ATMs leads to a 0.134 percent improvement in life expectancy, while a 1 percent upsurge in Debit cards tends to improve life expectancy by 0.091 percent. In the case of GMM models, findings infer that ATMs exert a significant and positive impact on life expectancy while Debit cards have no significant impact on life expectancy, as shown by statistically insignificant coefficient estimates of Debit cards. A coefficient estimate for ATMs displays that due to a 1 percent upsurge in FinTech, life expectancy tends to increase by 0.145 percent in Asian economies.

Our finding is consistent with (34), who infer that Fintech provides high-quality healthcare to the low-income and middle populations. Fintech is also directly facilitated society by reducing income inequality and poverty. Another possible reason is that Fintech improves green growth because Fintech fosters environmental quality and slows down climate change. This finding is also supported by Deng et al. (14), who noted that an inconclusive digital financial system supports sustainable development and plays a role in coherence with the social, economic, environmental, and governance. Meiling et al. (31) argued that Fintech encourages countries to achieve sustainable development without compromising social, economic, and environmental performance. Fintech is a modern pillar of the economy in the globe.

The digital revolution has fundamentally transformed Asian economies to develop new mechanisms for population health. Our results also support ICT diffusion theory Baliamoune-Lutz (35), which infers that Fintech reduces the cost of economic activities by reducing uncertainty in the health industry. This evidence is also consistent with earlier studies Klein (28) that Fintech supports sustainable growth and raises health sector industry standards in Asia. The impact of Fintech on health is clear in the COVID-19 pandemic (28). Fintech increases consumers' affordability of health care more efficiently in the digital economy. The health consequences of Fintech are significant, both in direct and indirect manners. Klein (28) also infers that Fintech has a larger indirect effect on health than indirect effects.

In the case of control variables, findings display that GDP results in improving life expectancy in two models, health expenditure tends to enhance life expectancy is only one model, internet results in improving life expectancy in three models, while FDI tends to improve life expectancy in all four models. The direct effects of GDP, health expenditure, ICT, and FDI are positive and significant on health, supporting the evidence of Dutta et al. (36), Nixon and Ulmann (37), and Strauss and Thomas (38).

## Conclusion and Implications

The progress of the health sector in a sustainable manner is crucial for the development of human capital, a significant and vital driver of economic growth. Many studies have argued that healthy workers are more productive, energetic, and more regular due to the absence of illness (1, 2). Therefore, to achieve macroeconomic stability and labor efficiency, the importance of the financial sustainability of the healthcare industry can't be ignored (3). The use of technology has transformed every sector of the economy, and the health sector is no exception. In this context, the amalgamation of technology into the financial industry known as financial technology (Fintech) has become crucial for the development health sector. Consistent with this view, we aim to investigate the impact of Fintech on health outcomes in Asian economies over the period 2007–2019. This analysis uses two proxies of health outcomes, including infant mortality and life expectancy. Similarly, to represent Fintech, we use different indicators such as ATMs, Debit cards, and the Internet.

The empirical estimation of the study is based on the 2SLS and GMM techniques. The outcomes confirmed the negative impact of ATMs and Debit cards on the infant mortality rate in both 2SLS and GMM models. Whereas, ATMs and Debit cards positively impact the life expectancy of people living in Asian economies irrespective of the estimation technique. Similarly, the association between the Internet and infant mortality rate is negative; whereas, this association is positive in the context of the Internet and life expectancy both with 2SLS and GMM. From these findings, we can confirm that the amalgamation of technology and the financial sector helps to improve health outcomes in Asian economies.

The analysis is crucial in providing guidelines to policymakers. The health sector is critical to developing human capital, a driver of sustainable economic growth. According to our findings, Fintech elevates the health status of emerging Asian economies. Therefore, the integration of Fintech into the health sector should be part and parcel of every health policy in emerging Asian economies. Digital finance can help the underprivileged faction of the society to save small amounts that can be withdrawn with profits at the time of health emergency. Hence, to uplift the health status of poor people, the focus of health policymakers should be on providing digital financial services to the poor and deprived people.

This study contains various limitations, such as it generally including ATMs and debit cards to measure the impact of financial technology on health outcomes. Future studies can also include other financial technology variables in the analysis. The author should also conduct a similar empirical analysis for other regions. Future researchers can estimate the asymmetric non-linear association between financial technology and health outcomes by adding other relevant variables.

## Data Availability Statement

Publicly available datasets were analyzed in this study. This data can be found at: https://data.worldbank.org/.

## Author Contributions

RJ and MH: conceptualization, software, data curation, and writing-original draft preparation. YM: methodology, writing-reviewing and editing. LZ: visualization and investigation. All authors contributed to the article and approved the submitted version.

## Funding

This study was supported by Research Project on Instructional Reform of Tertiary Education in Jilin Province Project name: Innovation and Practice of Diversified Talent Cultivation Mode in Financial Colleges and Universities under the Background of New Liberal Arts Construction (Project number: 20202855PO000D2) and Research on the promotion of the international status of Chinese standards (Grant No. 19QT008).

## Conflict of Interest

The authors declare that the research was conducted in the absence of any commercial or financial relationships that could be construed as a potential conflict of interest.

## Publisher's Note

All claims expressed in this article are solely those of the authors and do not necessarily represent those of their affiliated organizations, or those of the publisher, the editors and the reviewers. Any product that may be evaluated in this article, or claim that may be made by its manufacturer, is not guaranteed or endorsed by the publisher.

## Appendix

**TABLE A1:** Sample countries.

**No**	**Country**	**No**	**Country**
1	Bangladesh	10	Singapore
2	India	11	Thailand
3	Pakistan	12	Vietnam
4	Sri Lanka	13	Korea, Rep.
5	China	14	Russian Federation
6	Mongolia	15	Kazakhstan
7	Indonesia	16	Kyrgyz Republic
8	Malaysia	17	Tajikistan
9	Philippines	18	Uzbekistan
